# Non-Hereditary Obesity Type Networks and New Drug Targets: An In Silico Approach

**DOI:** 10.3390/ijms25147684

**Published:** 2024-07-12

**Authors:** Styliani A. Geronikolou, Athanasia Pavlopoulou, Merve Uça Apaydin, Konstantinos Albanopoulos, Dennis V. Cokkinos, George Chrousos

**Affiliations:** 1 Clinical, Translational Research and Experimental Surgery Centre, Biomedical Research Foundation of the Academy of Athens, 4, Soranou Ephessiou Str., 11527 Athens, Greece; dcokkinos@bioacademy.gr (D.V.C.); chrousosge@med.uoa.gr (G.C.); 2 University Research Institute of Maternal and Child Health and Precision Medicine, National and Kapodistrian University of Athens Medical School, Levadias 8, 11527 Athens, Greece; 3 Izmir Biomedicine and Genome Center (IBG), 35340 Izmir, Türkiye; athanasia.pavlopoulou@deu.edu.tr (A.P.);; 4 Izmir International Biomedicine and Genome Institute, Genomics and Molecular Biotechnology Department, Dokuz Eylül University, 35340 Izmir, Türkiye; 5 Iaso General Hospital, 15562 Athens, Greece; albanopoulos_kostis@yahoo.gr

**Keywords:** stress system/inflammation-induced obesity, stress-induced obesity, kisspeptin, natural products, systems epidemiology, drug discovery

## Abstract

Obesity, a chronic, preventable disease, has significant comorbidities that are associated with a great human and financial cost for society. The aim of the present work is to reconstruct the interactomes of non-hereditary obesity to highlight recent advances of its pathogenesis, and discover potential therapeutic targets. Obesity and biological-clock-related genes and/or gene products were extracted from the biomedical literature databases PubMed, GeneCards and OMIM. Their interactions were investigated using STRING v11.0 (a database of known and predicted physical and indirect associations among genes/proteins), and a high confidence interaction score of >0.7 was set. We also applied virtual screening to discover natural compounds targeting obesity- and circadian-clock-associated proteins. Two updated and comprehensive interactomes, the (a) stress- and (b) inflammation-induced obesidomes involving 85 and 93 gene/gene products of known and/or predicted interactions with an average node degree of 9.41 and 10.8, respectively, were produced. Moreover, 15 of these were common between the two non-hereditary entities, namely, ADIPOQ, ADRB2/3, CCK, CRH, CXCL8, FOS, GCG, GNRH1, IGF1, INS, LEP, MC4R, NPY and POMC, while phelligridin E, a natural product, may function as a potent FOX1-DBD interaction blocker. Molecular networks may contribute to the understanding of the integrated regulation of energy balance/obesity pathogenesis and may associate chronopharmacology schemes with natural products.

## 1. Introduction

Obesity is a complicated and often preventable chronic morbid entity of prioritized public health interest [1,2]. Contrary to common belief, it is an ancient disease, as shown in the medical history literature and archaeological excavation findings [3,4]. Although there have been time and cultural variations, over the ages, there have been described four main facets of obesity: rarity, gender dimorphism (females more frequently than males), increase with wealth and increase with modernization [5,6].

Currently, this chronic disease’s prevalence is increasing, reaching pandemic heights in Western societies. In 2022, the World Health Organization global estimates reported 2.5 billion overweight adults (Body Mass Index (BMI): 25–29), with 890 million adult subjects suffering from obesity (BMI ≥ 30) [7]. Thus, 16% of the planet’s adult population, or 67% of the population in the Americas and/or 19.6% of the Greek population, are overweight [7]. Over the past decades, obesity prevalence amongst children and adolescents has increased from 8% to 20%; 390 million subjects aged 5–19 years were overweight or obese at planet level, while 22 million children under 5 years were overweight [7]. From the above, 22 million, corresponding to 8% of the children and adolescents, were overweight and obese in 2022 [7]. In Greece, the prevalence rises to 41% in pubescent children and 28% in 15–16-year-old adolescents [7].

More importantly, it is expected that the prevalence of chronic diseases will increase accordingly as this particular morbid entity (obesity) intertwines with other maladies like diabetes, cardiometabolic syndrome, etc. [8,9].

Recent studies have established that body physiology is also regulated by an internal clock, resulting in functional oscillations of various rhythms (circadian, ultradian, infradian, etc.). These periodicities are encoded by certain genes (i.e., CLOCK, NR1D1/2, etc.) [10]. This point of view is underexplored, little known and worth investigating in metabolic disorder—as obesity—studies. Another vicious cycle is that of disturbed sleep, the stress system and metabolism [11,12,13]. Indeed, metabolic complications may originate from anxiety and/or stress [14,15]. Stress endemicity is a characteristic of Western societies in our times [15]. Stress triggered either by endocrine adjustments, due to pubertal evolution, or diverse emotional reactions to facing unexpected challenges characterizes, in particular, the adolescence developmental stage [16,17]. Obesity intertwines with body homeostasis and energy disturbance, favoring cardiovascular event development. On the other hand, with an ongoing emphasis on genetic and molecular approaches, oxidative stress has emerged as the dominant etiological mechanism of the disease, whilst investigation of anti-oxidative genes is of great research interest [18]. To this end, we previously generated an obesity interactions network (“obesidome”) [19], where we detected three origins of this chronic disease: stress-induced obesity, autonomic nervous system (ANS)/inflammation-induced obesity and genetic obesity [19]. We recently provided an update on the hereditary obesity molecular network specifically [20], highlighting the key role of kisspeptin in hereditary metabolism and obesity (genetic obesity). Accordingly, this study is focused in non-hereditary obesity (that is, obesity not of genetic or epigenetic origin).

Translational as well as post-translational research outcomes suggesting substantial complexity have been validated in human clinical trials [21,22]. Increased Body Mass Index is one of the main perils for non-communicable diseases (i.e., diabetes mellitus, musculoskeletal disorders, non-alcoholic fatty liver, obstructive sleep apnea, certain cancer types, cardiovascular diseases, infertility, depression), as well as for life expectancy and quality of life [23,24]. The mediating parameters of non-hereditary obesity in humans (energy expenditure, beige adipose tissue, oxidative stress, etc.) are of particular importance and need further research [2,23,24,25]. As credible clinical trials demand considerable cost, time and human resources of high expertise and specialization, in silico studies might save the time and costs of feasibility studies. A systems-level approach to obesity has been suggested previously as a pivotal need [26]. Therefore, the present study aims to a. provide an insight into the implications of inflammation, the autonomic nervous system and stress in metabolism and obesity pathogenesis, considering modern approaches such as circadian rhythms, and b. detect favorable drug targets and discover drug-like natural compounds.

## 2. Results

### 2.1. Stress-Induced Obesidome

**Figure 1:**
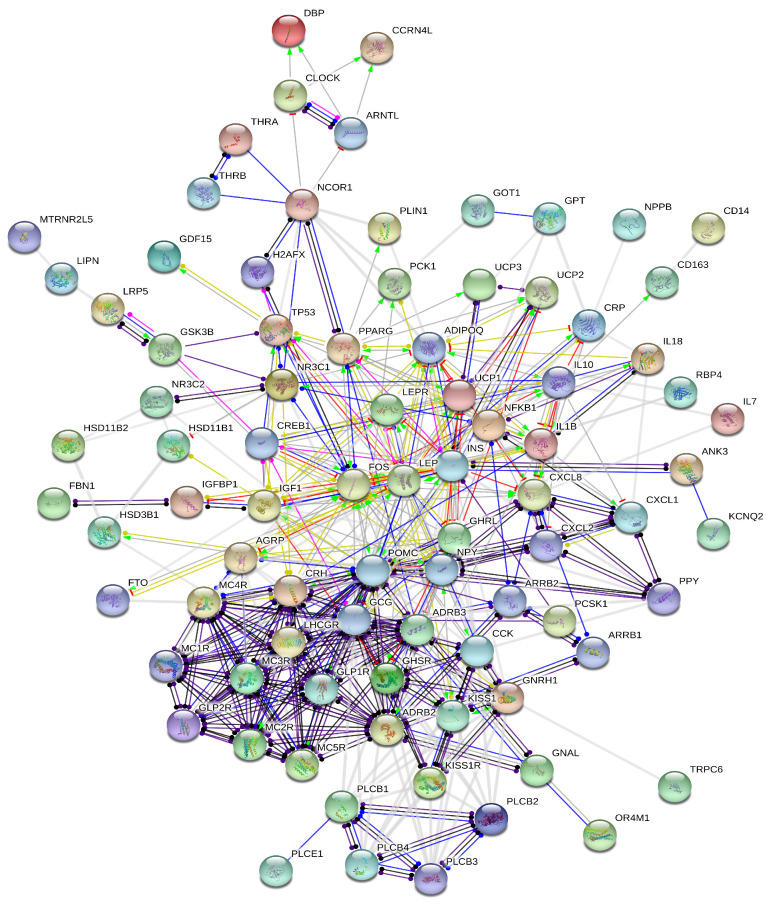
The updated interactome of stress-induced obesity, including the kisspeptin interactions network.

A network of associations of genes and/or gene products was generated (Figure 1) which consists of the 85 nodes listed in Table 1. This network is rather dense, with an average node degree of 9.41, suggesting that its constituent molecules strongly interact both physically and functionally. We identified 16 highly connected nodes (‘hubs’), where GCG, POMC, INS, LEP, NPY, CRH, FOS, ADIPOQ, MC4R, ADRB2, CXCL8, ADRB3, CCK, GHRL, GNRH1 and IGF1 have the highest number of connections (Table 1 and Table 2).

The above constructed interactome involves the kisspeptin-mediated network, which consists of fourteen nodes that represent the following genes/proteins (Figure 1): kisspeptin (KISS1) and its receptor (KISS1R), leptin (LEP), arrestin beta 1–2 (ARRB1/2), phospholipase C beta 1–4 (PLCB1–4), gonadotropin-releasing hormone 1 (GNRH1), transient receptor potential cation channel subfamily C member 6 (TRPC6), potassium voltage-gated channel subfamily Q member 2 (KCNQ2), phospholipase C epsilon 1 (PLCE1) and the luteinizing hormone/choriogonadotropin receptor (LHCGR). This network has been previously described and illustrated [20].

**Figure 2:**
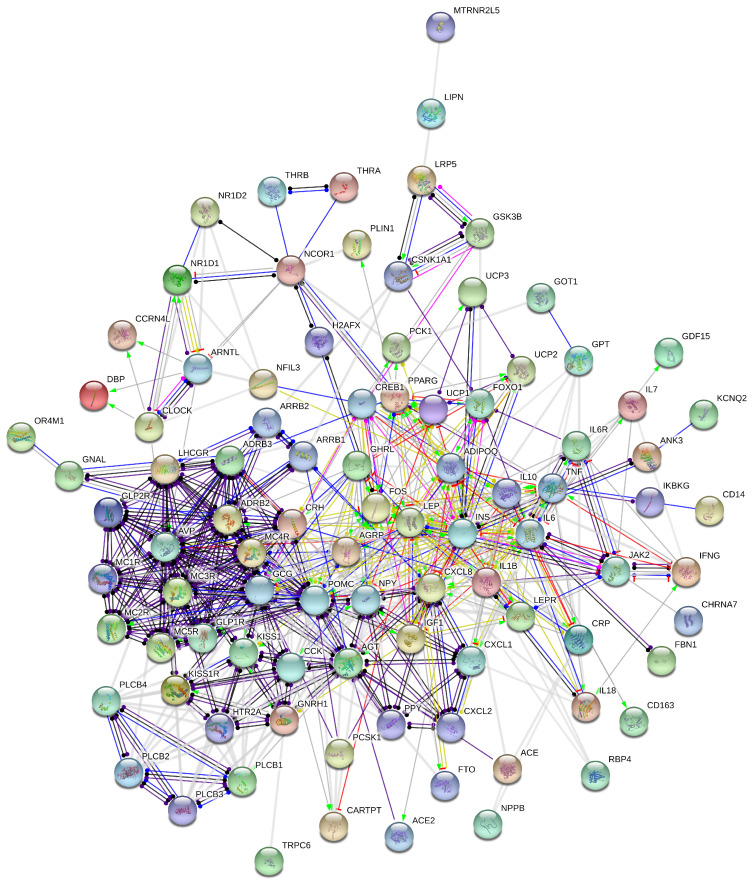
The interactome of ANS/inflammation-induced obesity, including the kisspeptin interactions network, asprosin interactions and circadian clock genes.

Kisspeptin interacts with components of its own network and also interacts directly and robustly with components of the stress-induced obesity network (Figure 2). KISS1 and KISS1R are connected directly to the ghrelin receptor (GHSR) and cholecystokinin (CCK) and, indirectly, via gonadotropin-releasing hormone 1 (GNRH1), to the corticotropin-releasing hormone (CRH) of the stress-induced obesidome. Notably, it is indirectly linked to leptin through GHSR.

**Figure 3:**
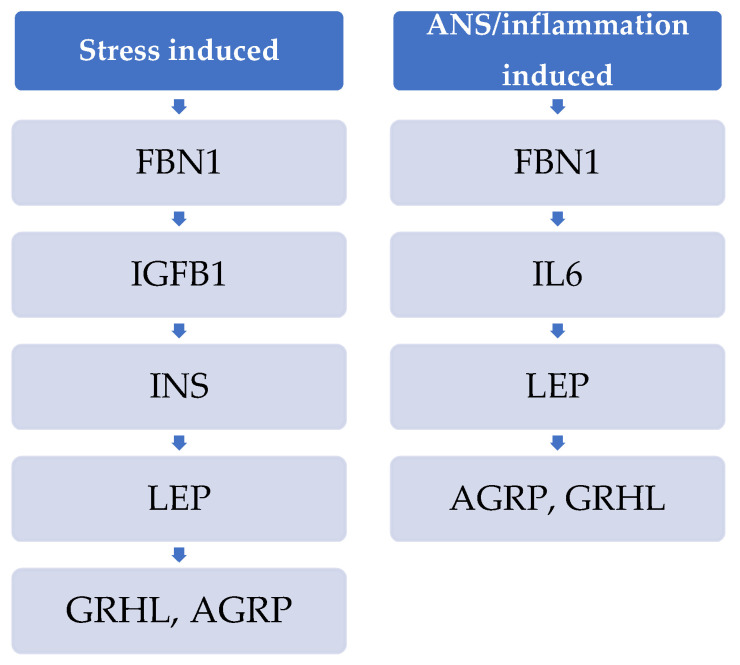
Asprosin signaling pathways in non-hereditary obesidomes.

The fibrilin 1 (FBN1) gene, which encodes the asprosin hormone, interacts only with IGFBP1 in the stress-induced obesity network (Figure 1 and Figure 3).

This network involves genes that have been associated with the circadian clock: CLOCK, ARNTL, DBP, CCRN4L, NR1D1/2 and NR3C1/2.

The created network involves 85 genes in total, listed in Table 1. The major hubs of the generated interactome are listed in Table 2.

### 2.2. Autonomic Nervous System/Inflammation Obesidome

A network of associations of gene and/or gene products was generated (Figure 2) that involves 93 nodes, indexed in Table 1, including the kisspeptin network. This network is rather solid, with an average node degree of 10.8, suggesting that the involved molecules strongly interact both physically and functionally. We identified 21 highly connected nodes (‘hubs’): INS, GCG, LEP, POMC, IL6, AVP, NPY, AGT, CRH, FOS, ADIPOQ, CXCL8, ADRB2, CCK, MC4R, GNRH1, IL10, ADRB3, IL1B, TNF and IGF1 (Figure 2, Table 2).

In the ANS/inflammation-induced obesity network, the fibrilin 1 (FBN1) gene, encoding the asprosin hormone, interacts only with IL6, which, in turn, interacts with interferon gamma (IFNG) (Figure 3).

This network comprises genes that have been linked to the circadian clock, namely, CLOCK, ARNTL, DBP, CCRN4L, NR1D1/2 and NR3C1/2. On the other hand, the CLOCK gene interacts with ARTNL, DBP, CCRN4L and NR1D1 and not with NCOR1 as in the stress-induced interactome.

### 2.3. Virtual Screening on Natural Compounds for Drug Discovery

**Figure 4:**
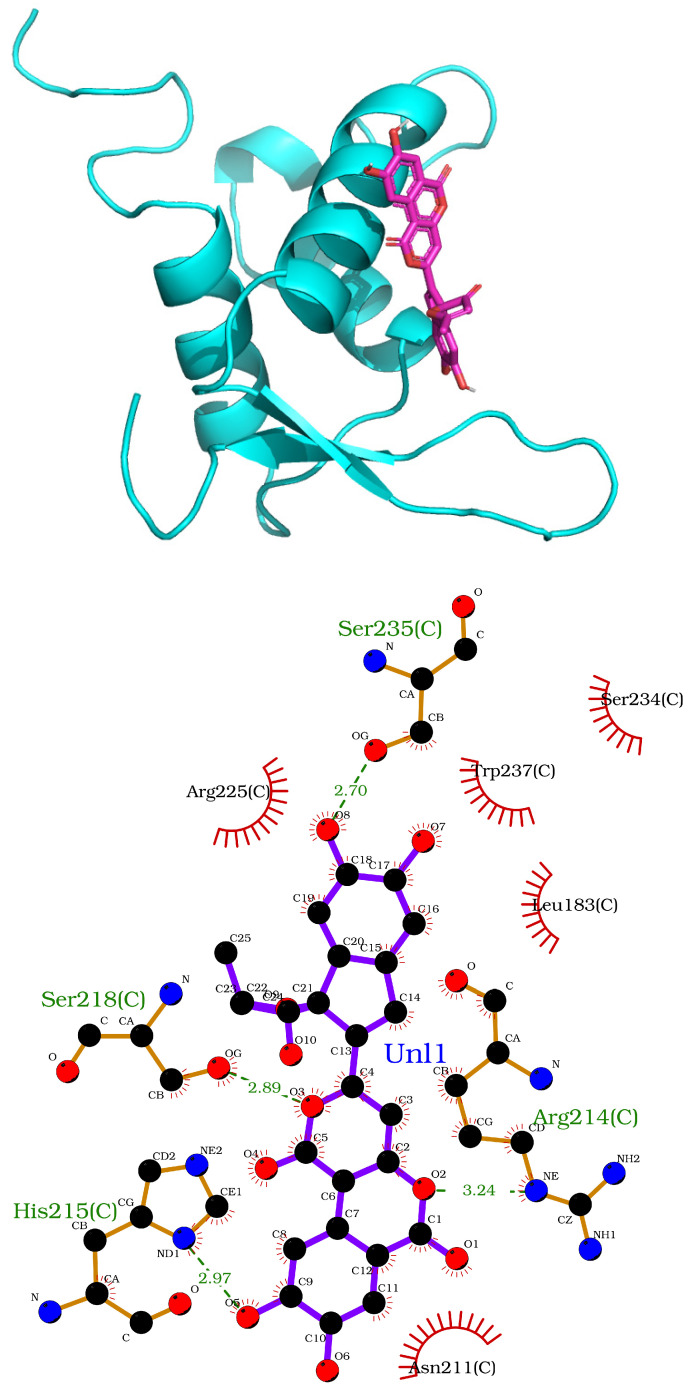
FOXO1–phelligridin E binding poses. Top: cartoon representation of FOXO1 and stick representation of phelligridin E. Bottom: 2D view of the phelligridin E binding sites.

By applying stringent criteria and parameters, we identified four chemical compounds with drug-like properties exhibiting a strong binding affinity to the FOXO1 protein (Table 3). These compounds are found in species of the birch family (carpinontriol B, alnusonol, acerogenin E) as well as the edible willow bracket fungus (phelligridin E). Members of the birch family have been shown to possess lipid-lowering properties [27,28,29]. Moreover, Wang and colleagues suggested that extracts of the willow bracket mushroom can inhibit lipase activity [30]. The amino acids Arg214, His215, Ser218 and Ser235 in the DBD of FOXO1 [31] appear to interact with phelligridin E (Figure 4) based on LigPlot2. According to NCBI CDD, these amino acids are implicated in DNA binding, suggesting a critical role of these residues in establishing FOXO1-DNA interaction. Phelligridin E could block this interaction by interfering with the DNA binding sites.

## 3. Discussion

The father of Western medicine, Hippocrates, in his selection “Hippocratic Corpus”, described obesity as deviation from the norm and, thus, a disease origin [4,32]. This balance was perceived as “harmony” by Pythagoras and referred to as “Eucrasia” after Hippocrates, as “Eustatheia” after Epicurus and, more recently, as “Homeostasis” by Walter Cannon [33]. All these terms mirror the balance in mind and body functions when adjusting to perpetually changing extrinsic challenges. Accordingly, the homeostasis principle governs every physiology structure, function and purpose. Disturbances of the homeostatic mechanisms intertwine with environmental stimuli perceived as stress by the human body; stress coordinates the body’s response to real, or perceived as such, extrinsic stimuli [34]. The adjusted homeostasis is maintained after stress system activation and peripheral and behavioral changes, resulting in the organism’s survival [34]. The homeostatic theory of obesity states that homeostasis preserves equilibrium through feedback loops for optimum body functioning. Long-lasting deviation from this balance (caused by extrinsic or intrinsic factors) triggers illness and well-being deprivation [35]. This theory links health (as defined by the WHO) with the understanding of illness (obesity herein), prevention and public health measures (socioeconomic, lifestyle, etc.), and, surely, to P4 medicine [36]. Our study attempted to decipher this process at the molecular level, contributing to P4 medicine [37].

Hereditary obesity includes the genetic obesity syndromes as well as the epigenetic obesity-related changes since the latter may be acquired by life challenges or lifestyle but are inherited to the descendants [19,20,38].

Conversely and notably, non-hereditary obesity originates from stimulatory lifestyle and environmental factors (‘obesogens’) [39,40].

### 3.1. Non-Hereditary Obesity Interactions Networks

The non-hereditary obesity presented herein extends to two different molecular and functional networks to depict and support the identification of novel insights and interpret the physiology implicated, and even attempts to suggest treatment options.

The corticotropin-releasing hormone (CRH) and locus ceruleus–norepinephrine/autonomic (LC/NE) systems and their peripheral effectors, the hypothalamic–pituitary–adrenal (HPA) axis and the limbs of the autonomic system comprise the principal components of the so-called stress system. The CRH and LC/NE systems dynamize arousal and attention, together with the mesocorticolimbic–dopaminergic system [41]. The latter is implicated in anticipatory and reward phenomena, whilst the amygdala generates emotions and fear [41]. Hypothalamic CRH mediates the inhibition of gonadotropin-releasing hormone secretion during stress. At the same time, it inhibits growth hormone through somatostatin, thyrotropin-releasing hormone and thyrotropin secretion as well. This way, it may suppress reproduction, growth and thyroid function. Glucocorticoids directly inhibit pituitary gonadotropin, growth hormone and thyrotropin secretion, rendering the relevant tissues of sex steroids and growth factors resistant to these substances [42]. Moreover, glucocorticoids trigger hepatic gluconeogenesis, and inhibit or generate an insulin effect on skeletal muscle and adipose tissue, respectively, thus promoting visceral adiposity and the metabolic syndrome [42]. Glucocorticoids may also act on the osseous matter, suppressing osteoblastic activity and hence inducing osteoporosis. More interestingly, patients with obesity suffering from mental disorders, either from anxiety with perception of ‘uncontrollable’ stress or from melancholic depression, frequently manifest mild hypercortisolism. The latter induces a sequela of misfunctions driving to diverse morbid entities such as stress-induced glucocorticoid-mediated visceral obesity and metabolic syndrome clinical signs, etc. [43]. At the end of this chain, a manifold increase might occur in all-cause mortality risk to affected subjects, driving a curtailing of their life expectancy [44]. Glucocorticoids are secreted in a circadian manner. The ways the suppressed corticoid oscillations result in obesity and hormone dynamics influence adipogenesis are stillf under investigation. Irregular glucocorticoid profiles may result from aberrant sleep and feed schedules, metabolic syndrome, prolonged glucocorticoids administration or chronic stress [45].

As shown in Figure 2, the circadian genes implicated in the stress-induced obesity are depicted as interacting in the following ways: CLOCK interacts with ARTNL and DBP. ARTNL interacts directly with DBP, CCRN4L and CLOCK. NR3C1 interacts with FOS, TP53, GSK3B, CRH and ADIPOQ. CCRN4L is linked to ARNTL and CLOCK. More importantly, FOXO1, CSNK1A1, NF1L3 and NR1D1 clock genes/proteins are not involved in this type of obesity.

Beyond stress, inflammation is also an innate immunity response to extrinsic (infective or occasionally noninfective) or intrinsic (dysfunctional) stimulating factors that aims to maintain homeostasis, which, when it is chronic, may become detrimental [44,46]. In the overweight and obesity cases, inflammation interweaves with them. Proinflammatory factors (e.g., NFKB) and five of the main markers of chronic inflammation (IL10, IL7, IL1B, IL6 and TNF) are involved in this interaction network created herein (Figure 3). TNFα is a pleotropic molecule implicated in apoptosis, insulin signaling, lipid metabolism, inflammation and immune system involvement [47]. Its levels in peripheral blood increase with weight gain and decrease with weight loss [48]. It further triggers apoptosis of adipocytes and insulin resistance through insulin receptor suppression, oxidation and oxidative phosphoryliosis in adipocytes’ mitochondria [49].

Concerning the circadian clock, in the ANS/inflammation-induced obesity network constructed herein, ARNTL interacts with CLOCK, NR1D1 and CCRN4L. The latter is linked to both CLOCK and ARNTL. CLOCK interacts with DBP, apart from NR1D1, CCRN4L and ARNTL. CLOCK and ARNTL disturbance may trigger obesity, hyperinsulemia and diabetes [50,51,52,53].

DBP interacts only with ARNTL and CLOCK. Notably, FOXO1 lies upstream of CSNK1A1, PPARG, CSK3B, IGF1, CREB1, PCK1, LEP, ADIPOQ and AGRP. The biological clock regulates metabolism by driving transcriptional patterns (i.e., the nuclear hormone receptors NR1D1/2), the same as CCRN4, controlling directly the transcription of certain rate-limiting enzymes for cholesterol and fatty acids metabolism and insulin sensitivity [54]. In turn, PPARG and other mitochondrial-rated enzymes are shown to be governed by FOXO1 in this type of obesity and not in stress-induced obesity. Thus, the FOXO1 circadian-related gene is revealed as a key drug target in ANS/inflammation-induced obese (weight-loss-demanding) patients.

Translational and clinical research has established that the trafficking of immune cells (monocytes, neutrophils, T and B lymphocytes) follows circadian patterns. More interestingly, IL7 secretion is promoted by glucocorticoids in an α diurnal base, supporting T cell distribution and, thus, immune response to various soluble antigens or bacterial infections at night [55]. It has been evidenced that the sequel of an infection pivots not only on the pathogen (parasitic, bacterial or viral), but also on the time of the day it initiated [56,57,58,59,60,61]. These studies have shown that the genes ARNTL (mainly), DBP and CLOCK strongly modulate the magnitude of the infections. The infections, in turn, trigger a circadian rhythm amplitude decrease [62,63], although the mechanism of host–pathogen interactions requires further elucidation.

The commensal intestine microbiota of our intestine and its implication in metabolism and obesity have been major research targets during the last two decades. Disturbance of clock genes repeals rhythms in the intestine microbiota [64]. NFIL3 regulates the expression of a circadian lipid metabolic program and modulates lipid absorption in intestinal epithelial cells [63]. Our research showed that NFIL3—involved only in the ANS/inflammation interactome—interacts with CLOCK, ARNTL1, NR1D1/2, IL10 and CREB1. IL10 is a key anti-inflammatory cytokine that may suppress the proinflammatory responses of adaptive as well as innate immunity [65,66,67]. Both immunities are rhythmicity dependent, known to be guided by ARNTL1 and NR1D1/2 [68,69,70]. Finally, CREB1 has been assessed as a non-pharmacological strategy against obesity and associated diseases [71,72].

This finding suggests that the rhythmicity of the gut microbiota’s role in metabolism needs urgently further elucidation.

Our molecular interactions networks mirror the above-stated pathways of stress-induced obesity (Figure 1, Table 1), as well as of autonomic nervous system inflammation-induced obesity (Figure 2, Table 1). The created interactomes comprise genes, transcription factors, receptors, enzymes, gut hormones, autocrine and paracrine hormones of the hypothalamus, pancreas and liver, lipid-gated channels of the cell membrane, kinases, etc. They are highly connected networks, suggesting functional interactions among the aforementioned molecules.

The number of major hubs of the created interactions networks is 16 for the stress-induced obesity and 21 for the inflammation/ANS obesidome (Table 2). Thirteen of these highly connected molecules are common between the two networks: ADIPOQ, ADRB2, ADRB3, CCK, CRH, FOS, GCG, GNRH1, INS, LEP, MC4R, NPY and POMC. More interestingly, AGT, AVP, CXCL8, IL6, IL10 and IL1B are additional and exclusive major hubs in the inflammation/ANS obesidome, whilst GHRL is the extra highly coupled hub in the stress-induced obesidome (Table 2).

Leptin induces cholecystokinin gene expression, synthesizing the gastrointestinal hormone cholecystokinin in the first segment of the small intestine (duodenum). In the stress-induced obesity network, CCK inhibits NPY, which regulates the feeding behavior [19,73]. Moreover, LEP regulates the satiety factor CART, which, in turn, is implicated in the release of extracellular-signal-regulated kinases inside the cell [19].

The autonomic nervous system is a key player in the integrated short-term regulation of weight, regulating the satiety signal and energy expenditure [74]. The afferent vagal pathways seem to dominate between the gut and the brain, acting in a composite way over gut hormones. Sympathetic nervous system physiology involves lipolysis stimulation and energy expenditure via sympathetic innervation of white and/or brown adipose tissue [74,75]. In obesity, this function dysregulates in a compensatory, though ineffective, fashion, that is, sympathetic stimulation may induce the development of hypertension, organ damage or even overt cardiovascular disease [74].

Although preliminary clinical trials investigating autonomic modulation as a treatment for obesity provided opposing results, mechanistic and pathophysiology studies strongly support this therapeutic strategy as an appealing and promising treatment for obesity [74,76]. Moreover, in a previous work of ours, we distinguished the bariatric surgery technique’s feasibility according to the ANS activity and patient’s clinical history [77]. Of particular interest, in the stress-induced obesity, HTR2A interacts with CCK and GHSR.

The autonomic initiation involves CRH, as explained above, activating POMC, MC1/2/3/4/5R, ADRB2-3 and IL6 pathways. Furthermore, CHRNA7 (cholinergic receptor nicotinic alpha 7 subunit) is shown to stimulate JAK2, which, in its turn, interacts with LEP, IL6R and the neurotransmitter HTR2A (serotonin receptor 2A) [78]. In our networks, POMC interacts with MC4R, glucagon, leptin, ADRB3, MC2R and 21 more nodes. It is connected to kisspeptin directly via leptin, or indirectly via other nodes.

The exclusively highly connected nodes of genes encoding β adrenergic receptors in ANS/inflammation-induced obesity highlight the tight link between thermoregulation (i.e., metabolism) and the autonomic/immunity systems. The thermogenic regulation of adipose tissue is mediated by noradrenaline release, hence triggering β adrenergic receptors and fostering brown adipocyte proliferation [79]. Animal studies attempted to validate the thermoregulation dysfunction complexity [80,81] with opposing results. Subsequently, further efforts were directed towards controlling cold-induced thermogenic pathway dysregulation and obesity through β adrenergic receptor agonist synthesis and administration [82]. Clinical and translational studies provided evidence regarding the involvement of ADRB2 and ADRB3 in body mass regulation. Studies in different human populations evidenced that known polymorphisms of *ADRB2* and *ADRB3* were associated with receptor inhibition and weight gain [83]. More interestingly, Kurylowicz et al. detected lower ADRB2 and ADRB3 gene expression in visceral adipose tissue (VAT) than in subcutaneous adipose tissue (SAT) in bariatric surgery patients [84]. This finding, together with no methylation differences assessed in SAT and VAT, suggests a decreased lipolytic potential of VAT. Apart from these studies, our interactomes corroborate previous epidemiological reviews suggesting that “subcutaneous fat exerts a lower risk of metabolic complications than visceral fat” [84].

The kisspeptin network has been previously described [20]. Kisspeptin (encoded by the *KISS1* gene in humans) is a peptide with pleotropic activity that is implicated in puberty and in polycystic ovary syndrome onset [16,17], as well as in diverse homeostatic mechanisms with anti-oxidative effect and glucose homeostasis among them. Thus, as oxidative stress is the main pathogenic mechanism of chronic stress linked to the obesity epidemic, kisspeptin emerges as a target of interest for the obesity-oriented research. Furthermore, kisspeptin is implicated in metabolic and reproductive functions that crosstalk in many ways [85,86].

**Figure 5:**
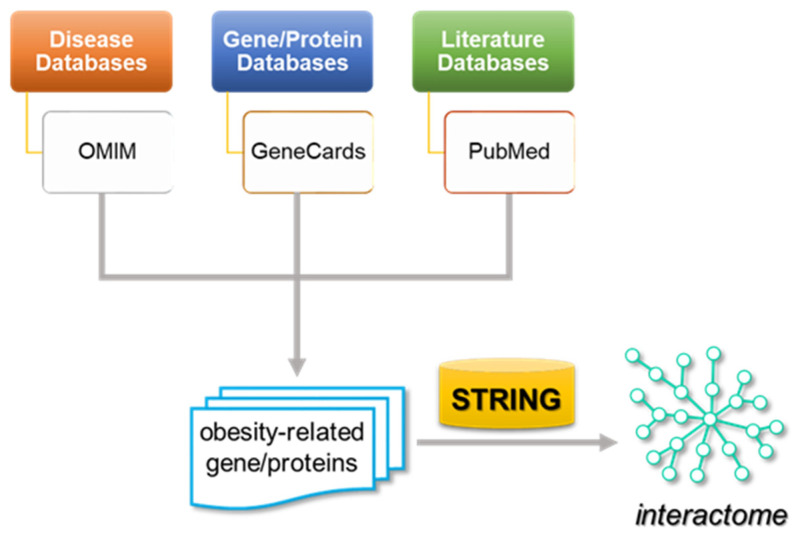
Workflow chart illustrating the methodology followed in this study.

Asprosin and its encoded gene (*FBN1*) are research targets of interest in animal and human studies. While its role in obesity is controversial, it is established that it is implicated in the pathophysiology of cancer, cardiovascular diseases and possibly metabolic diseases (i.e., diabetes, polycystic ovary syndrome) [87]. Our interactomes elucidate novel interactions of FBN1, through which non-hereditary types of obesity may result. More interestingly, FBN1 interacts directly with IFNG in stress-induced obesity but with IL6 in ANS/inflammation-induced obesity. Indeed, “asprosin is able to bind to the Toll-like receptor 4 (TLR4) through a TLR4/JNK-mediated pathway to increase ROS production and pro-inflammatory cytokines, thereby promoting inflammation and apoptosis of β-cells” [87,88]. Its role in the ANS has not been elucidated yet, although it has been suggested that the central receptors of asprosin located in the hypothalamic arcuate nucleus may contribute to appetite promotion (and, thus, energy excess and obesity), through the anorexigenic pro-opiomelanocortin (POMC) neurons and the orexigenic agouti-related peptide (AgRP) neurons [87,89,90]. Our molecular networking approach overhauls this hypothesis by identifying differential physiology routes both in stress-induced obesity and in ANS/inflammation-induced obesity (Figure 5). In turn, IFNG is activated by cortisol, the strongest known stress marker [87].

Our interactomes illustrate the metabolic roles of FOXO1, asprosin and kisspeptin, providing evidence for further research by pointing them out as drug target candidates.

This study is limited to entire genes/gene products and the data available in the specific databases (based on experimental evidence). The confidence interactions score was set at >0.97; thus, interactions with a lower score are not included or discussed. Our work highlighted recent advances in obesity physiology and expanded our understanding of the daily pathophysiology of the disease. Furthermore, our networks elucidated novel pathways and/or molecular interactions that could even contribute to chronopharmacology repurposing. The latter is pivotal for all aspects of P4 medicine.

### 3.2. Potent Natural Compounds Targeting FOXO1

We previously identified GLP1 as a major hub of the obesidome. GLP1 has been a popular drug target with spectacular effects on diabetes II control, but became a weight loss pharmacological option in the last decade. Diabetes drugs (GLP1 agonists) mimic GLP1 action, stimulating insulin production after glucose increase due to feeding. Dulaglutide (Trulicity) (weekly), Semaglutide (Ozempic) (weekly), Exenatide extended release (Bydureon bcise) (weekly), Exenatide (Byetta) (twice daily), Liraglutide (Victoza, Saxenda) (daily), Lixisenatide (Adlyxin) (daily) and Semaglutide (Rybelsus) (taken by mouth once daily) are medicaments that target GLP1 and are widely prescribed for this purpose.

Our interactomes revealed new drug targets of interest for each type of obesity. In our effort to discover in silico potential drug-like compounds, we found that it was not feasible for most of them. In particular, asprosin protein is not druggable as it contains a series of tandem repeats of the calcium-binding epidermal growth factor (EGF)-like domain [91]. Kisspetin analogs have been suggested for metabolic disorders and fatty liver control [92], emerging as research targets of interest for many groups. Thus, we opted to focus on the FOXO1 circadian clock gene, which regulates all the other interactions as it is upstream of them (Figure 3).

Through an in silico approach, we identified four active principles from natural products (Table 3) that may inhibit the FOXO1 circadian clock gene’s obesogenic activity. Of those, phelligridin E (Figure 4) has been tested for its antimicrobial properties [93] in human cell lines and rodents. Cell lines have been modeled for identifying the anti-oxidant activity of carpinontriol B, acerogenin E and alnusonol as well [94,95]. None of these substances has been investigated for its activity on weight gain or loss, or been checked for toxicity. Yet hazelnut is known for its lipid-lowering properties [96,97]. In ethnic/traditional medicine, hornbeam is suggested for its anti-diabetic/obesity activity [27], Japanese alder for fatty liver control (http://www.stuartxchange.org/CompleteList.html; accessed on 18 June 2024) and willow bracket fungus for its anti-tumor/alcoholic liver/inflammatory/diabetic properties. Accordingly, birch is known for its action against gynoid lipodystrophy (herniated subcutaneous fat within fibrous connective tissue). These compounds, which can be isolated from natural resources [98] in a cost-effective way, represent drug candidates and merit further investigation.

## 4. Methods

The overall methodology followed in the present study is depicted in Figure 5.

### 4.1. Network Construction

The biomedical literature database PubMed/MEDLINE (https://www.ncbi.nlm.nih.gov/pubmed accessed on 29 April 2024) was searched manually for scientific studies relevant to biological clock, stress, autonomic nervous system and obesity-related genes or gene products (up to 30 January 2023) [19,85]. The collected articles were thoroughly read, and the relevant terms were retrieved. The publicly accessible databases OMIM (https://omim.org/ accessed on 29 April 2024) and GeneCards (https://www.genecards.org/ accessed on 29 April 2024) were searched subsequently. A network-based approach was employed to investigate the interactions among the retrieved genes/gene products. To this end, the functional and physical associations among the genes/proteins were investigated and visualized through STRING v11.0 [99], a database of experimentally supported or predicted association data. A relatively high confidence interaction score (≥0.7) was applied. Of note, only those interactions derived from experimental studies, deep-learning-based text mining of the scientific literature and knowledge databases of curated protein complexes/pathways were included in order to minimize any false-positive associations. The methodology followed in this study is illustrated in Figure 1.

### 4.2. Drug Discovery

We considered kisspeptin, asprosin and FOXO1 as putative drug targets. The DNA binding domain (DBD) [31] of the human FOXO1 protein was opted for as a potential target for drug discovery based on natural compounds. 

#### 4.2.1. High-Throughput Virtual Screening of FOXO1 Protein

##### Target Protein

The resolved three-dimensional (3D) structure of the DBD of the human FOXO1 protein bound to double-stranded DNA was obtained from the RCSB Protein Data Bank (PDB), San Francisco, CA, USA [100]; PDB ID: 3CO6.

##### Chemical Compound Screening

The Comprehensive Natural Products Database (COCONUT) [101], filtered based on satisfying Lipinski’s Rule of 5, and, having been retrieved from the Zinc database only, was used to compile a refined set of 43,129 natural compounds from diverse sources and chemical classifications. The 3D structures of the selected compounds were generated using the RDKit release 2024.03.1 (http://www.rdkit.org/; accessed on 29 April 2024) Python library. Additionally, partial charges and protonation states were incorporated for the newly constructed ligands to improve their structural quality. These chemical compounds were screened blindly against the FOXO1 tertiary structure to discover potential ligands.

##### Molecular Docking Simulations

The 43,129 compounds were subjected to a series of docking simulations on the FOXO1 protein. The docking analyses were conducted using smina [102], a robust docking engine and a fork of Autodock Vina [103]. These experiments were implemented within an in-house developed Nextflow [104] workflow (accessed on 7 May 2024) in order to integrate and automate the workflow. In this way, the binding interactions between the natural compounds and the FOXO1 protein were explored. Structure files and docking log files were obtained for each natural compound, capturing their top nine most stable conformers. By setting the binding affinity K_D_ value to be less than −9 kcal/mol, lead candidates were identified by assessing the docking scores of the best conformers for each compound with the FocO1 protein.

##### Molecular Visualization

The molecular images were generated with the PyMOL Molecular Graphics System, version 3.0, Schrodinger LLC (https://www.schrodinger.com/products/pymol; accessed on 3 May 2024). LigPlot2 was also used for the automatic generation of two-dimensional (2D) representations of the protein–ligand interactions [105].

#### 4.2.2. Protein Motif Detection

The amino acid sequence corresponding to the DBD of FOXO1 (UniProtKB accession number: Q12778) was scanned against the NCBI CDD database v3.21 [106] of annotated protein domains and functional sites to identify functionally important residues.

## 5. Conclusions

Both updated obesity networks reveal functional connections, providing a novel insight into the human body’s energy homeostasis by illustrating novel major “crossroads”. Our ANS/inflammation-induced obesidome corroborates previous findings, yielding to future drug treatment focused on FOX1 and adrenergic regulation and revealing the beta-adrenergic receptor’s key role, whilst the stress-induced network evidences the role of asprosin and kisspeptin as drug candidates for obesity. The circadian machinery presented herein provides a novel insight in terms of chronotherapy and the personalized approach to treating aspects of the disease. Thus, concerning ANS/inflammation anti-obesity, we identified phelligridin E (a natural product), which could block the FOX1-DBD interaction by interfering with the FOX1-DNA binding sites and constitute a key active substance of novel anti-obesity drug(s).

In future research, it is worth focusing on the following:

- Discovering new chronopharmacological drugs against obesity;
- Anticipating possible toxic effects of such natural products due to long-term taking;
- Ultradian and circadian rhythmicity identification and contribution to obesity pathogenesis as well as control;
- Through reverse translational research, repurposing not only drugs, but also pertinent biomarkers.

## Figures and Tables

**Table 1:** The updated stress-induced (1) and ANS/inflammation-induced (2) obesity interactions network.

Gene/Protein Symbol	Description	Network
ACE	angiotensin-converting enzyme	2
ACE2	angiotensin-converting enzyme 2	2
ADIPOQ	adiponectin, C1Q, and collagen domain containing	1, 2
ADRB2	adrenoceptor beta 2	1, 2
ADRB3	adrenoceptor beta 3	1, 2
AGRP	agouti-related neuropeptide	1,2
AGT	Angiotensinogen	2
ANK3	ankyrin 3	1, 2
ARNTL	basic helix–loop–helix ARNT-like 1	1,2
ARRB1	arrestin beta 1	1, 2
ARRB2	arrestin beta 2	1, 2
AVP	arginine vasopressin	2
CARTPT	cocaine- and amphetamine-regulated transcript	2
CCK	Cholecystokinin	1, 2
CCRN4L	nocturnin	1, 2
CD14	CD14 molecule	1, 2
CD163	CD163 molecule	1, 2
CHRNA7	neuronal acetylcholine receptor subunit alpha	2
CLOCK	nocturnin	1, 2
CREB1	cAMP responsive element binding protein 1	1, 2
CRH	corticotropin-releasing hormone	1, 2
CRP	C-reactive protein	1, 2
CSNK1A1	casein kinase 1 alpha 1	2
CXCL1	C-X-C motif chemokine ligand 1	1, 2
CXCL2	C-X-C motif chemokine ligand 2	1, 2
CXCL8	C-X-C motif chemokine ligand 8	1, 2
DBP	D-box binding PAR bZIP transcription factor	1, 2
FBN1	fibrillin 1	1,2
FOS	Fos proto-oncogene, AP-1 transcription factor subunit	1, 2
FOXO1	forkhead box O1	2
FTO	FTO alpha-ketoglutarate-dependent dioxygenase	1, 2
GCG	Glucagon	1, 2
GDF15	growth differentiation factor 15	1, 2
GHRL	Ghrelin	1, 2
GHSR	growth hormone secretagogue receptor	1
GLP1R	glucagon-like peptide 1 receptor	1, 2
GLP2R	glucagon-like peptide 2 receptor	1, 2
GNAL	G protein subunit alpha L	1,2
GNRH1	gonadotropin-releasing hormone 1	1, 2
GOT1	glutamic-oxaloacetic transaminase 1	1, 2
GPT	glutamic-pyruvic transaminase	1, 2
GSK3B	glycogen synthase kinase 3 beta	1, 2
H2AX	H2A.X variant histone	1, 2
HSD11B1	hydroxysteroid 11-Beta Dehydrogenase 1	1
HSD11B2	hydroxysteroid 11-Beta Dehydrogenase 2	1
HSD3B1	hydroxy-delta-5-steroid dehydrogenase, 3 beta-, and steroid delta-isomerase 1	1
HTR2A	5-HT2A receptor	2
IFNG	interferon gamma	2
IGF1	insulin-like growth factor 1	1, 2
IGFBP1	insulin-like growth factor binding protein 1	1
IKBKG	inhibitor of nuclear factor kappa B kinase regulatory subunit gamma	2
IL10	interleukin 10	1, 2
IL18	interleukin 18	1, 2
IL1B	interleukin 1 beta	1, 2
IL6	interleukin 6	2
IL6R	interleukin 6 receptor	2
IL7	interleukin 7	1, 2
INS	insulin	1, 2
JAK2	janus kinase 2	2
KCNQ2	potassium voltage-gated channel subfamily Q member 2	1, 2
KISS1	KiSS-1 metastasis-suppressor	1, 2
KISS1R	KISS1 receptor	1, 2
LEP	Leptin	1, 2
LEPR	leptin receptor	1, 2
LHCGR	luteinizing hormone/choriogonadotropin receptor	1, 2
LIPN	lipase family member N	1, 2
LRP5	LDL receptor related protein 5	1, 2
MC1R	melanocortin 1 receptor	1, 2
MC2R	melanocortin 2 receptor	1, 2
MC3R	melanocortin 3 receptor	1, 2
MC4R	melanocortin 4 receptor	1, 2
MC5R	melanocortin 5 receptor	1, 2
MTRNR2L5	MT-RNR2-like 5 (pseudogene)	1, 2
NCOR1	nuclear receptor corepressor 1	1, 2
NFIL3	nuclear factor, interleukin 3 regulated	2
NFKB1	nuclear factor kappa b	1
NPPB	natriuretic peptide B	1, 2
NPY	neuropeptide Y	1, 2
NR1D1	nuclear receptor subfamily 1 group D member 1	2
NR1D2	nuclear receptor subfamily 1 group D member 2	2
NR3C1	nuclear receptor subfamily 3 group C member 1	1
NR3C2	nuclear receptor subfamily 3 group C member 2	1
OR4M1	olfactory receptor family 4 subfamily M member 1	1, 2
PCK1	phosphoenolpyruvate carboxykinase 1	1, 2
PCSK1	proprotein convertase subtilisin/kexin type 1	1, 2
PLCB1	phospholipase C beta 1	1, 2
PLCB2	phospholipase C beta 2	1, 2
PLCB3	phospholipase C beta 3	1, 2
PLCB4	phospholipase C beta 4	1, 2
PLCE1	phospholipase C epsilon 1	1
PLIN1	perilipin 1	1, 2
POMC	proopiomelanocortin	1, 2
PPARG2	peroxisome proliferator-activated receptor gamma	1, 2
PPY	pancreatic polypeptide	1, 2
RBP4	retinol-binding protein 4	1, 2
THRA	thyroid hormone receptor alpha	1, 2
THRB	thyroid hormone receptor beta	1, 2
TNF	tumor necrosis factor	2
TP53	tumor protein p53	1
TRPC6	transient receptor potential cation channel subfamily C member 6	1, 2
UCP1	uncoupling protein 1	1, 2
UCP2	uncoupling protein 2	1, 2
UCP3	uncoupling protein 3	1, 2

**Table 2:** Major network hubs.

Stress Induced		ANS/Inflammation Induced	
Hub	Interactions	Hub	Interactions
GCG	32	INS	37
POMC	32	GCG	34
INS	31	LEP	34
LEP	29	POMC	32
NPY	25	IL6	29
CRH	22	AVP	28
FOS	22	NPY	27
ADIPOQ	19	AGT	25
MC4R	19	CRH	24
ADRB2	18	FOS	22
CXCL8	18	ADIPOQ	21
ADRB3	17	CXCL8	21
CCK	17	ADRB2	20
GHRL	17	CCK	20
GNRH1	17	MC4R	20
IGF1	17	GNRH1	19
		IL10	19
		ADRB3	18
		IL1B	18
		TNF	18
		IGF1	17

**Table 3:** Top-ranking natural compounds targeting FOXO1.connective.

COCONUT CID	Docking Scores (kcal/mol)	Compound Name	Source	2D Image
CNP0087111	−14.7	carpinontriol B	*Corylus avellana* (hazelnut); *Carpinus cordata* (hornbeam)	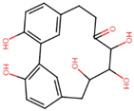
CNP0191412	−13.5	alnusonol	*Alnus japonica* (Japanese alder)	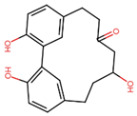
CNP0307211	−13	acerogenin E	*Betula* spp. (birch)	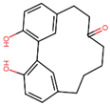
CNP0276542	−9.3	phelligridin E	*Phellinus igniarius* (willow bracket fungus)	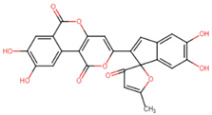

## Data Availability

No new data were created or analyzed in this study.
